# A Novel Approach to Localizing and Treating Post-surgical Hemobilia With Combined Radiofrequency Ablation and Biliary Stent Placement

**DOI:** 10.7759/cureus.83312

**Published:** 2025-05-01

**Authors:** William K Oelsner, James Laney, Kristie Liao, Arslan Kahloon, George Philips

**Affiliations:** 1 Department of Gastroenterology and Hepatology, University of Tennessee Health Science Center College of Medicine - Chattanooga, Chattanooga, USA; 2 Department of Internal Medicine, University of Tennessee Health Science Center College of Medicine - Chattanooga, Chattanooga, USA; 3 Division of Gastroenterology and Hepatology, Department of Internal Medicine, University of Michigan, Ann Arbor, USA

**Keywords:** endobiliary radiofrequency ablation, endoscopic retrograde cholangiopancreatography (ercp), hemobilia treatment, laparoscopic cholecystectomy complications, post-surgical hemobilia, spyglass cholangioscopy, upper gastrointestinal bleeding

## Abstract

Hemobilia is a rare but potentially life-threatening condition that has traditionally been treated with biliary stent placement. However, this method is often inadequate for localizing bleeding and is prone to recurrence. Recently, advances in cholangioscopy have offered both direct visualization of hemobilia and expanded treatment modalities. Here, we propose a novel approach to identifying and treating recurrent post-surgical hemobilia using cholangioscopy in conjunction with dual radiofrequency ablation (RFA) and biliary stent placement in a 78-year-old man who developed a biliary leak, intrahepatic infection, and recurrent hemobilia after laparoscopic cholecystectomy.

## Introduction

Hemobilia, bleeding into the hepatobiliary tract, is a rare but potentially life-threatening condition, which accounts for 6% of all upper gastrointestinal bleeds [1-3]. Disruptions of the normal architecture of the liver parenchyma and biliary tract, including trauma, infection, inflammation, vascular anomalies, and malignancy, may result in hemobilia; however, the most common cause is iatrogenic trauma from instrumentation [4,5]. Procedures such as percutaneous needle biopsy, endoscopic retrograde cholangiopancreatography (ERCP), choledochoscopy, biliary stent placement, sphincterotomy, percutaneous biliary drainage, and cholecystectomy have an inherent risk of iatrogenic hemobilia [4,6,7].

The management of hemobilia is centered on achieving hemostasis while preventing biliary obstruction. Historically, surgery for hemobilia has resulted in up to a 10%-12% mortality; however, advances in endoscopic and interventional radiology (IR) techniques have reduced mortality to 3.6% [3,8]. Specifically, advances in cholangioscopy offer the opportunity to directly visualize the bleed while allowing for therapeutic intervention. Several case reports have been published on the use of biliary stents to both tamponade the hemorrhage and allow biliary drainage [9-12]. For massive hemobilia, these interventions are temporizing until definitive treatment with surgery or interventional radiology can occur. Recently, two case reports demonstrated the effectiveness of endobiliary radiofrequency ablation (RFA) and stent placement for maintaining hemostasis for malignant hemobilia secondary to metastatic pancreatic cancer and hepatocellular carcinoma [13,14]. To our knowledge, the use of these modalities to treat post-surgical hemobilia has not been reported in the literature. Here, we present a novel approach for investigating and treating post-surgical hemobilia with cholangioscopy, RFA, and biliary stent placement.

## Case presentation

A 78-year-old Caucasian man with a history of atrial fibrillation on warfarin and osteoarthritis presented for an outpatient ERCP for evaluation of recurrent hemobilia over the past several months.

One year prior, the patient was admitted to the surgical intensive care unit (SICU) as a transfer from an outside facility following a bile duct leak complicated by severe sepsis and new upper gastrointestinal bleeding. The patient had initially undergone an outpatient cholecystectomy for biliary colic and was immediately discharged from the ambulatory surgery center. Five days post-procedure, the patient presented to an outside medical facility for worsening abdominal pain. He was found to have* E. coli* bacteremia with imaging findings concerning for a bile duct leak, prompting transfer for ERCP intervention. On arrival, the patient was hemodynamically stable and noted to have melena. He underwent a diagnostic laparotomy with abdominal washout and drain placement, followed by ERCP. During endoscopy, he was noted to have active hemobilia at the ampulla and a cholangiogram with common bile duct (CBD) filling defects, which were identified as clotted material after sweeping the duct. The patient was also found to have extravasation of contrast adjacent to the cystic duct clips, confirming a Strasberg type A bile leak. Hemobilia and the biliary leak were treated with biliary stent placement. A post-procedure interventional radiology angiography failed to localize hemorrhage or identify pseudoaneurysm formation on two separate occasions. During this hospitalization, the patient required multiple blood transfusions for acute blood loss anemia complicated by hypotension.

One month following discharge, the patient re-presented with septic shock due to *E. coli* bacteremia complicated by intrahepatic liver abscesses, which were medically managed. During this hospitalization, there was no evidence of gastrointestinal bleeding. On hospital discharge, the patient was placed on warfarin for new atrial fibrillation.

**Figure 1:**
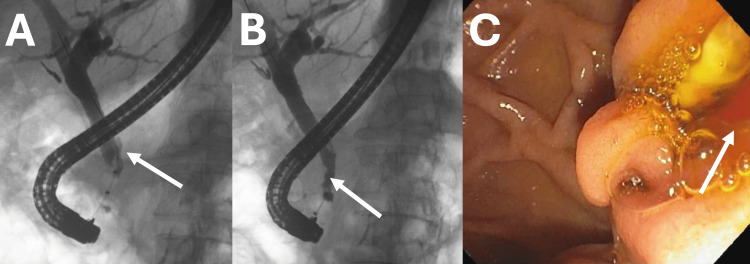
Cholangiogram with type III cystic duct take-off (A) and adjacent common bile duct filling defect (B). During completion of cholangioscopy, bright red blood (C) was noted to be coming out of the papilla on direct endoscopic visualization.

Over the past several months, the patient reported continued intermittent episodes of melena, concerning for ongoing hemobilia. The patient was scheduled for outpatient ERCP with cholangioscopy for further evaluation. On cholangiogram, a small filling defect was noted in the mid-common bile duct, and during occlusion cholangiography, bright red blood was observed coming out of the ampulla (Figure 1).

**Figure 2:**
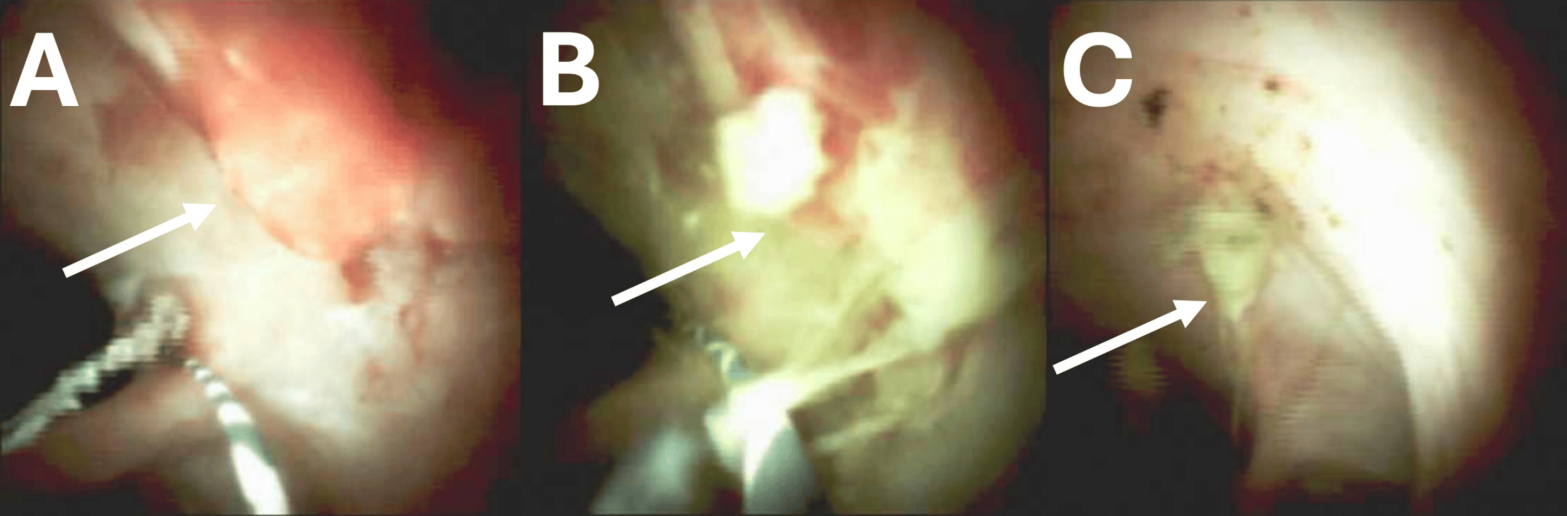
Visible vessel at the junction of the cystic remnant and common bile duct before (A) and after (B) RFA. Follow-up cholangioscopy performed three months post-treatment with residual RFA artifact (C) and resolution of recurrent hemobilia. RFA: radiofrequency ablation

At this point, it was decided to use the cholangioscope for direct visualization. The scope was advanced over the guidewire to the hilum. During withdrawal, a visible vessel was discovered at the junction of the cystic remnant and the CBD (Figure 2A). Given the severity of previous bleeds with the need for anticoagulation for atrial fibrillation, we elected to perform endobiliary radiofrequency ablation (RFA) with the Habib endobiliary RFA catheter (Boston Scientific, Marlborough, MA) for two 60-second cycles. Prior to the procedure, the patient and his wife were counseled and provided informed consent for treatment with this therapeutic approach. The cholangioscope was exchanged back, and ablation of the vessel was visually confirmed (Figure 2B). A 10 × 80 mm covered metal stent was deployed for added tamponade and prevention of stricture development post-RFA treatment.

The RFA procedure was successful, and the patient underwent biliary stent extraction during a three-month follow-up ERCP. At that time, resolution of the visible vessel following combined RFA and biliary stent treatment was confirmed by direct cholangioscopy (Figure 2C). Five years following treatment, there were no hospitalizations for recurrent upper gastrointestinal bleeding and hemobilia.

## Discussion

Hemobilia following laparoscopic cholecystectomy is a rare complication and is thought to stem from vascular irritation from bile leak and infection, leading to pseudoaneurysm formation [7,15,16]. Our patient had multiple reasons for developing hemobilia, including biloma formation and intrahepatic infection secondary to bile leak following a Clavien-Dindo grade III event. What makes this case unique compared to prior case reports is our approach to both the investigation of hemobilia and intervention to maintain durable hemostasis.

Unless there is a clinical suspicion for hemobilia, it is often difficult to diagnose on esophagogastroduodenoscopy (EGD) or side-viewing duodenoscopy given the intermittent nature of the hemorrhage. Furthermore, ERCP requires the presence of amorphous or tubular cast-like filling defects to suggest hemobilia in the right clinical context [15]. Currently, imaging with helical computed tomographic angiography (CTA) and magnetic resonance cholangiopancreatography (MRCP) can confirm the diagnosis; however, these modalities are limited in that they offer localization without therapy. Recently, cholangioscopy has provided an avenue for both detecting hemobilia and localizing hemorrhage in hemodynamically stable patients [17]. In this case, the synergistic use of cholangioscopy in conjunction with RFA and stent placement allows for both the localization and treatment of hemobilia.

The treatment of hemobilia ranges from conservative management for stable patients to IR embolization and surgery for unstable patients. Intrabiliary application of hemostatic agents during ERCP has not been studied, given the risk for biliary obstruction. Recently, endobiliary stent placement has been shown to be successful in tamponading hemobilia in several case reports, although this has yet to be formally studied [4,15]. Now, endobiliary radiofrequency ablation in conjunction with stent placement is emerging as a repurposed modality for treating malignant hemobilia. Linz et al. (2017) [13] and Kim et al. (2002) [14] illustrated the utility of RFA and biliary stent placement for treating hemobilia due to pancreatic cancer and hepatocellular carcinoma. While there is a paucity of studies into the use of cholangioscopy and RFA for hemostasis of hemobilia, it does pose an interesting modality for both localization and treatment. One hurdle for the adoption of this emerging technology is that few medical centers have access to cholangioscopy and intrabiliary radiofrequency ablation equipment or advanced endoscopists trained in their use. Further studies need to be conducted on the efficacy and safety of using RFA and cholangioscopy for hemobilia before widespread use.

## Conclusions

Here, we present a novel approach for localizing and treating post-surgical hemobilia with cholangioscopy in conjunction with endobiliary radiofrequency ablation and stent placement. Hemobilia following laparoscopic cholecystectomy is a rare complication, and to our knowledge, no prior report has been published on the use of radiofrequency ablation in the treatment of post-surgical hemobilia. This case illustrates the potential for using cholangioscopy as a modality for both investigating and treating benign post-surgical hemobilia.
